# The neural basis of swap errors in working memory

**DOI:** 10.1073/pnas.2401032121

**Published:** 2024-08-05

**Authors:** Matteo Alleman, Matthew Panichello, Timothy J. Buschman, W. Jeffrey Johnston

**Affiliations:** ^a^Department of Neuroscience, Center for Theoretical Neuroscience and Zuckerman Mind, Brain, and Behavior Institute, Columbia University, New York, NY 10027; ^b^Department of Neurobiology, Stanford University, Stanford, CA 94305; ^c^Princeton Neuroscience Institute and Department of Psychology, Princeton University, Princeton, NJ 08544

**Keywords:** neural population, working memory, swap errors

## Abstract

When making decisions in a cluttered world, humans and other animals often have to hold multiple items in memory at once—such as the different items on a shopping list. Psychophysical experiments in humans and other animals have shown remembered stimuli can sometimes become confused, with participants reporting chimeric stimuli composed of features from different stimuli. In particular, subjects will often make “swap errors” where they misattribute a feature from one object as belonging to another object. While swap errors have been described behaviorally and theoretical explanations have been proposed, their neural mechanisms are unknown. Here, we elucidate these neural mechanisms by analyzing neural population recordings from monkeys performing two multistimulus working memory tasks. In these tasks, monkeys were cued to report the color of an item that either was previously shown at a corresponding location or will be shown at the corresponding location. Animals made swap errors in both tasks. In the neural data, we find evidence that the neural correlates of swap errors emerged when correctly remembered information is selected from working memory. This led to a representation of the distractor color as if it were the target color, underlying the eventual swap error. We did not find consistent evidence that swap errors arose from misinterpretation of the cue or errors during encoding or storage in working memory. These results provide evidence that swap errors emerge during selection of correctly remembered information from working memory, and highlight this selection as a crucial—yet surprisingly brittle—neural process.

## Significance

Working memory is central to cognition. It allows us to remember multiple items at once, and use those items to guide future behavior—such as remembering the items on a grocery list. However, working memory is brittle. Not only are items forgotten or remembered imprecisely, they also get mixed up with each other in “swap” or “misbinding” errors. For example, setting out to buy red apples and green pears from the store, but ending up with green apples and red pears. Interestingly, we show that these errors emerge from the misselection of correctly remembered items from working memory—indicating that working memory may be sharply limited by our ability to reliably manipulate rather than simply represent multiple distinct items.

Whether recalling items on a shopping list, or matching new names to faces, reckoning with the limits of one’s working memory is an inescapable part of human experience. Behavioral studies have characterized limits in the storage and selection of multiple items from working memory (1–5). For instance, when subjects are cued to report one of the features (e.g., the color) of a target item from a set of remembered items, the likelihood of an error is increased by either increasing the number of items that must be remembered (3, 4, 6) or decreasing the time for which the items are presented (7, 8).

Previous work has provided insight into the neural mechanisms that lead to the drift of memories over time and the “forgetting” of items (9, 10), but a third type of error—the “swap error”—is not well understood. Swap errors occur when a subject reports a feature value that was present among the remembered items but that does not correspond to the target item (4, 11). For example, if a red square and blue circle are shown to a subject, when asked what color the square had been the subject might make a swap error and report “blue.”

Swap errors have been of particular interest not only because they provide insight into fundamental limitations of working memory (12, 13), but also because they may arise from failures to correctly bind the distinct features of a particular stimulus together (14–18). In the previous example, the subject might say the square was blue because they actually remember seeing a blue square and red circle. However, the relationship of swap errors to binding has been controversial, as different explanations—such as an error when interpreting or representing the cue (19) or incomplete encoding of the stimulus array (20)—can give rise to the same behavioral pattern. For instance, the subject might mishear the experimenter and say blue because they thought they were supposed to report the color of the circle. While recent work has shown that swap errors are reflected in neural activity after the target is known but before the response period (21)—and thus do not reflect a last-minute guess—existing work leaves many possible explanations for swap errors open.

To address the controversy around the nature of swap errors, we analyzed neural population recordings from macaque monkeys performing a color working memory task. Consistent with previous work in humans, on a subset of trials monkeys made swap errors by incorrectly reporting the color of the distractor (rather than the target). Surprisingly, we found evidence that swap errors occurred during the selection of correctly remembered information from working memory. We replicated this pattern of results in a second attention task, which also elicited swap errors and had a similar neural mechanism. Overall, this work provides insight into the neural mechanisms underlying widely observed limitations in working memory—and highlights the fragility of manipulating information in working memory.

## Results

**Fig. 1.**
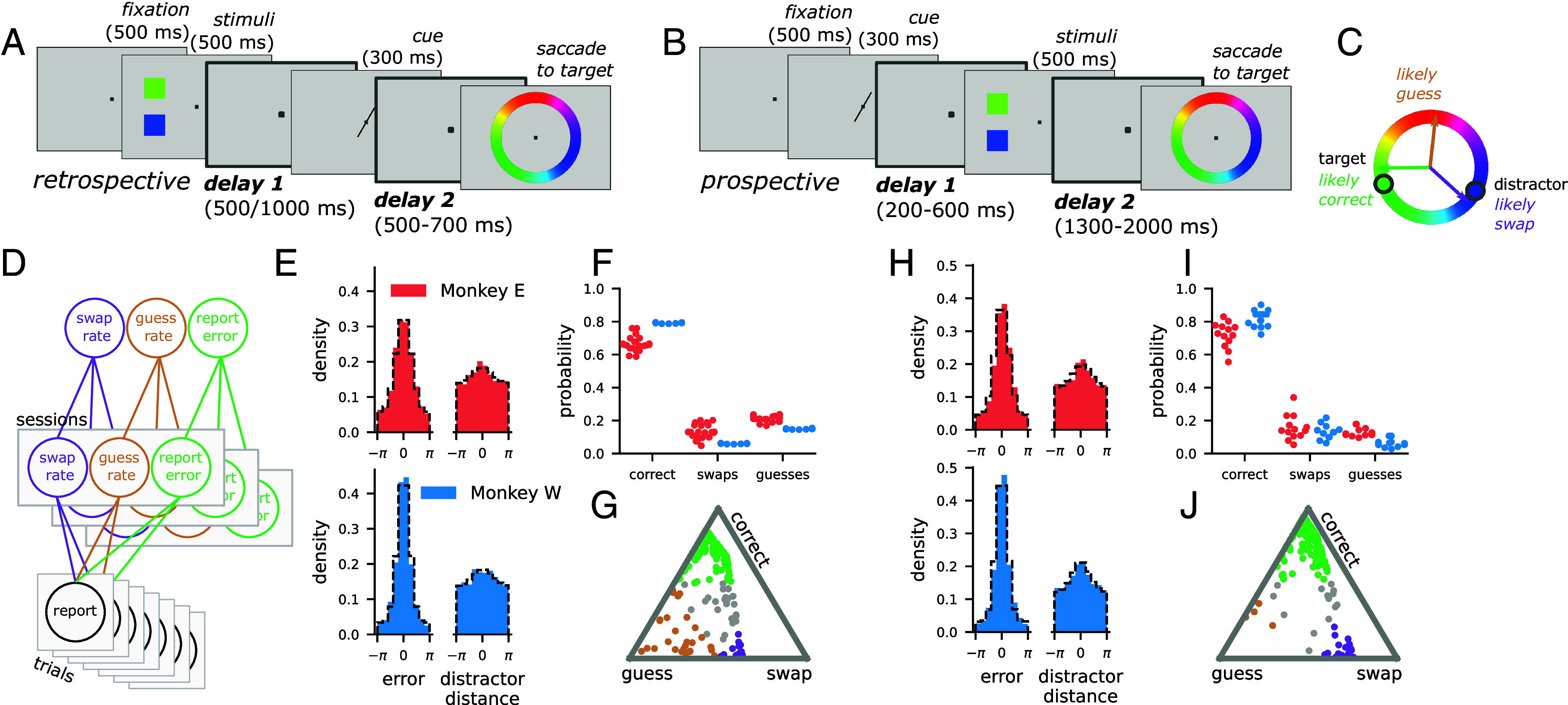
The retrospectively and prospectively cued working memory tasks, as well as the behavioral modeling approach. (*A*) Schematic of the retrospective task. (*B*) Schematic of the prospective task, note that the order of the cue and stimuli is switched with respect to the respective task shown in (*A*). (*C*) Schematic of the possible trial outcomes: correct response (close to the target), a guess (uniform random), and a swap (close to the distractor). (*D*) Schematic of the behavioral modeling approach. Each trial response is explained in terms of three parameters: the rate of swap errors, the rate of guesses, and the dispersion around the target response. A single model is fit hierarchically across all the sessions, where the session level parameters (*Middle*) are modeled as being sampled from a distribution of animal-level parameters (*Top*). The model is fit separately for each animal. (*E*) Histograms of the errors from the target (*Left*) and the distractor stimulus (*Right*) for Monkey E (*Top*) and Monkey W (*Bottom*) on retrospective trials. The dashed black line is the posterior predictive distribution from the behavioral model. The concentration around zero for the plots on the *Right* indicates that both monkeys make swap errors. (*F*) The inferred rate of each response type on retrospective trials for each session (points) and both monkeys (colors). (*G*) An example session from Monkey E on the retrospective task. Each point is the response on a particular trial. The triangle is the three-dimensional simplex for the probabilities of each response type. Each of the three corners represent probability 1 of a particular response type. Colored points have probability >0.5 of belonging to their respective response type. (*H*–*J*) The same as (*E*–*G*) but for prospective trials, and the example session in (*J*) is from Monkey W.

Monkeys performed two versions of a continuous working memory task (Fig. 1 *A* and *B*) (22). In the “retrospective” task, the animal remembered two colors, one presented at an upper spatial position (the upper color) and one presented at a lower spatial position (the lower color; Fig. 1*A*). After a memory delay period, an abstract cue reliably indicated whether the color at the upper or lower position was the “target.” After a second memory delay, the animal reported the color of the target by making a saccade to the matching color on a color wheel. Note, the color wheel was rotated on every trial to prevent motor planning. Therefore, the retrospective task required the animal to hold two color-position pairs in working memory before selecting one, and reporting the corresponding color. Monkeys also performed a “prospective” task which was identical with the exception that the cue was shown before the colors (Fig. 1*B*). In this condition, the monkey could use visual attention to select the relevant target stimulus immediately and ignore the other “distractor” stimulus (23, 24). Previous work with this task has shown that the neural representations of the items in working memory are distributed across the prefrontal, parietal, and visual cortex in both tasks (22). It also showed that the retrospective selection and prospective attention conditions were surprisingly similar: In both cases, the representation of the target stimulus was dynamically transformed—in the same way—to facilitate the animal’s behavioral response (22).

### Both Behavioral Tasks Elicit Swap Errors.

In the tasks, the animals made three qualitatively different kinds of responses (Fig. 1*C*): correct responses; guess responses, where the animal chooses a random color; and swap responses, where, as introduced above, the animal reports the color of the wrong (distractor) memory. Following the human working memory literature (4) (but see ref. 25), we model the distribution of the behavioral responses within each task (Fig. 1 *E* and *H*) as arising from a mixture model with probabilities for each response type (Fig. 1*D*, and see *The behavioral model*). The generative model produced by the fit closely matched the empirical distribution of animal responses (Fig. 1 *E* and *H*, black dashed-line compared to colored histogram).

This model shows significant evidence for all three types of response across both the retrospective and prospective tasks. In the retrospective task, both monkeys performed the majority of trials correctly (Monkey E: correct probability = 0.60 to 0.72, Monkey W: correct probability = 0.76 to 0.82, unless otherwise noted all reported ranges are 95% CIs for the relevant quantity; Fig. 1 *E* and *F*) but also made guess (Monkey E: guess probability = 0.16 to 0.27, Monkey W: guess probability = 0.12 to 0.18) and swap errors (Monkey E: swap probability = 0.08 to 0.19; Monkey W: swap probability = 0.04 to 0.08). There was a similar pattern in the prospective task (Fig. 1 *H* and *I*, Monkey E: correct probability = 0.65 to 0.78, guess probability = 0.09 to 0.18, swap probability = 0.10 to 0.22; Monkey W: correct probability = 0.73 to 0.85, guess probability = 0.03 to 0.13, swap probability = 0.09 to 0.19). While the behavior of monkey E was variable from session to session in both tasks (Monkey E: mean swap rate across sessions = 0.11 to 0.15 in the retrospective task, and mean swap rate across sessions = 0.12 to 0.20 in the prospective task), monkey W was variable in the prospective task only (Monkey W: mean swap rate across sessions = 0.11 to 0.16), with highly consistent behavior in the retrospective task (Monkey W: mean swap rate across sessions = 0.06 to 0.06). This result indicates that while the rate at which monkey W makes swap errors fluctuates from session to session in the prospective task, a single constant swap rate provides the most likely explanation of their behavior on the retrospective task. This difference can be revealed by our behavioral model due to its hierarchical structure, which assumes that the session-level swap rates are drawn from a distribution for which we fit the mean and variance. Using the fitted model, we computed the posterior probability that a particular trial arose from each response type (Fig. 1 *G* and *J*, *Bottom*), which we incorporated into our probabilistic model of the neural population data below.

### Neural Correlates of Swap Errors during Encoding and Maintenance of Stimuli in Working Memory.

We recorded single and multiunit neural activity simultaneously from posterior parietal cortex, frontal cortex, motor cortex, posterior inferotemporal cortex, and visual area 4 (posterior parietal cortex: 10 to 39 units; motor cortex: 18 to 53 units; prefrontal cortex: 22 to 57 units; V4 and posterior IT: 15 to 33 units; frontal eye fields: 2 to 23 units, where the ranges give the minimum and maximum number of simultaneously recorded neurons across sessions). Since swap errors are rare and each specific trial configuration was also rare, we were unable to combine the neural populations across sessions. Thus, to increase our statistical power, we took advantage of the distributed nature of working memory representations (22, 26) and analyzed all of the units recorded in a single session as a single population, combining across the different brain regions (combined: 80 to 181 units). We performed a region exclusion analysis to determine whether the observed effects depended only on neural activity from specific regions; this analysis found no consistent effects across monkeys (*SI Appendix*, *Region–dropping analysis* and Fig. S1).

**Fig. 2.**
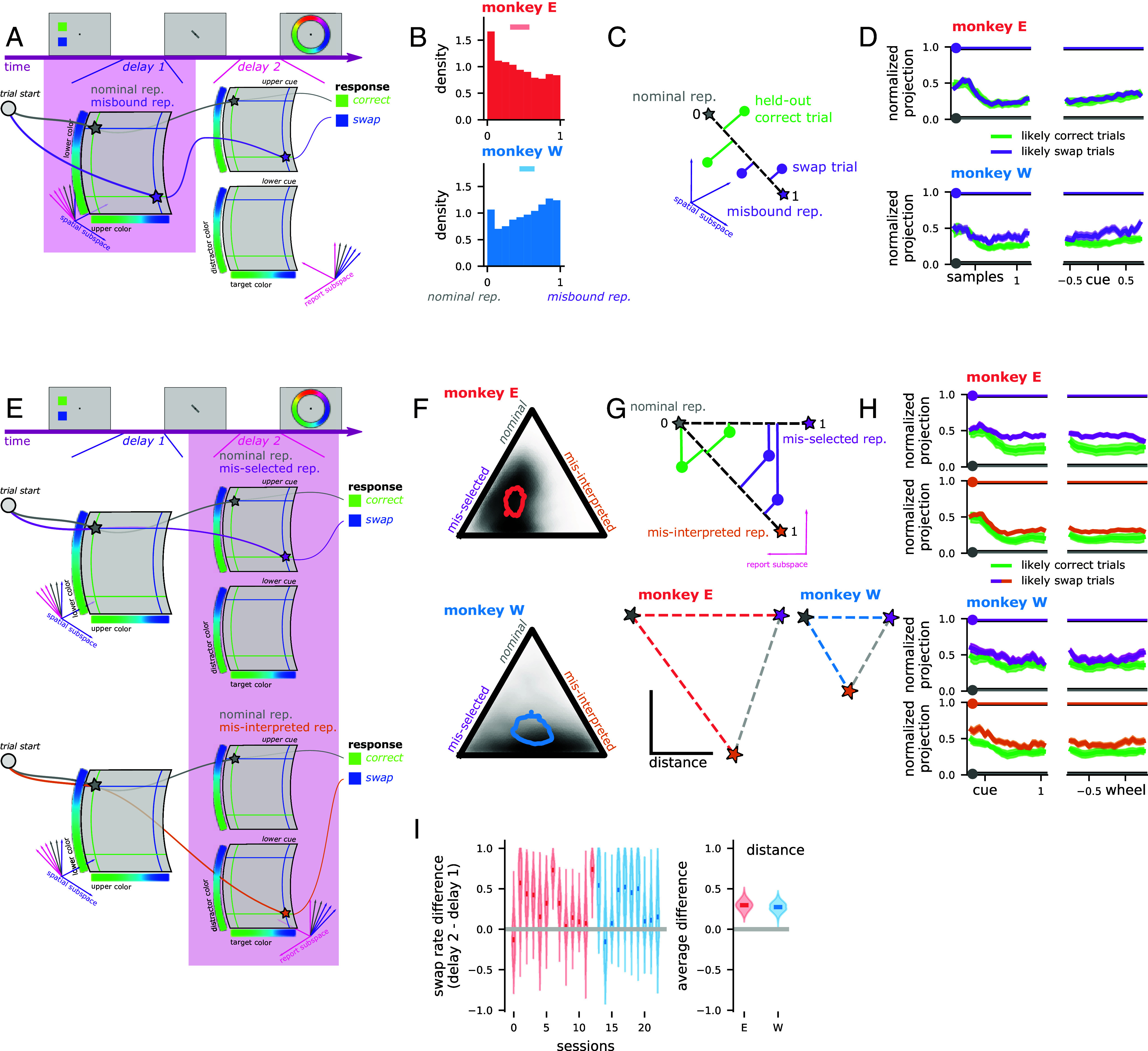
The neural correlates of swap errors in the retrospectively cued working memory task. (*A*) Schematic of an example trial during the first delay period of the retrospective task. The nominal (gray star) and misbound (purple star) representations are shown for this example trial. The misbound representation manifests as a swapped representation of the upper and lower colors (green-upper, blue-lower to blue-upper, and green-lower). (*B*) The average (*Top* bar) and aggregate posterior (distribution) across sessions for the $\;p_{\;\text{misbind}}\;$ parameter in the neural mixture model for the first delay period. Values close to one indicate an association between swap errors and misbound representations; values close to zero indicate that trials with swap errors are still associated with the nominal representation. (*C*) Schematic of the cross-validated version of the analysis. Nominal and misbound representations (stars) are constructed for each trial using the same kind of linear model as used in the neural mixture model. The model is fit only on likely correct trials. Then, held-out correct trials and swap trials (circles) are projected onto the dimension connecting the nominal and misbound representations (dashed line). The distance is normalized to be between zero and one. (*D*) Time course of evidence for an association between swap errors and the misbound representation under the analysis in (*C*), for each monkey (*Top* and *Bottom*) as well as centered on the time of sample (*Left*) and cue (*Right*) presentation. (*E*) Schematic of the same example trial from above, showing the contrast between the nominal representation (gray stars) and the misselected colors representation (*Top*, pink star) as well as the misinterpreted cue representation (*Bottom*, orange star). (*F*) The average (contour outline) and aggregate posterior (heatmap) evidence for the nominal, misselected colors, and misinterpreted cue representations under the neural mixture model in delay 2. (*G*) (*Top*) Schematic of the cross-validated version of the analysis. Correct, color selection, and cue interpretation prototypes are constructed using a linear model fit on likely correct trials. Then, held-out correct trials and likely swap trials are projected along the axes connecting the nominal representation with each of the error representations and normalized as before. (*Bottom*) The estimated average geometry of these three hypothesized representations, all distances are significantly greater than zero for both monkeys. (*H*) Time course of evidence for the misselected colors and misinterpreted cue representations for both monkeys (*Top* and *Bottom*) as well as centered on the cue presentation (*Left*) and appearance of the response wheel (*Right*). (*I*) The difference between the evidence for an association between swap errors and the alternative representations in delay 1 and delay 2 under the neural mixture model for each session (*Left*) and averaged across all sessions (*Right*; positive values indicate more evidence for correlates in delay 2).

We then compared the neural activity on trials with correct or swap responses to the neural representation that would be expected if the color of each item were faithfully represented (“nominal rep.,” Fig. 2*A*, gray star) and several alternate neural representation (e.g., the right colors represented in the wrong positions; Fig. 2*A*, purple star). In all analyses, we first preprocessed the data by z-scoring and then performing principal components analysis to retain 95% of the variance (Monkey E: average 106 dimensions retained after preprocessing; Monkey W: 88). We begin by analyzing the first delay period of the retrospective task (Fig. 2*A*). In this period, the monkey must remember both the two colors and their locations; so, we expect that both colors will be represented in the neural population activity. In particular, we fit the neural activity with the linear model,

[1] $$\begin{array}{cc} r(\;u\;,\;l\;) & =W_{u}f\left(\;u\;\right)+W_{l}f\left(\;l\;\right)+\eta \end{array}$$

where $W_{u}$ and $W_{l}$ are fitted matrices, u and l are the colors presented in the upper and lower positions, respectively, f(·) is a function that transforms the color (given in radians) to a representation in spline basis functions (*The neural mixture model*), and η is noise. Given a green upper color and a blue lower color, the nominal neural representation is $\;r_{\;\text{nominal}}\;=r\left(\;\text{green},\;\text{blue}\right)$. One alternate neural representation that we consider here is a misbound representation, $\;r_{\;\text{misbinding}}\;=r\left(\;\text{blue},\;\text{green}\right)$, where color and position are misassociated. That is, if behavioral swap errors arise from a misbinding between color and spatial position, then we expect the neural representation on trials with behavioral swap errors to resemble the misbound representation $\;r_{\;\text{misbinding}}\;$ (Fig. 2*A*, “misbound rep”) rather than the nominal representation $\;r_{\;\text{nominal}}\;$. On trials with correct behavioral responses, we expect that the neural representation will resemble the nominal representation (Fig. 2*A*, “nominal rep.”).

We express our hypotheses about neural representations in a mixture model, with a parameter controlling the probability that the neural activity is sampled from a distribution around the misbound representation instead of the nominal representation. This approach allows us to avoid a hard categorization of trials into correct and swap responses. Instead, we use the posterior probability that a trial had a correct or swap response under the full behavioral model. For an observed response ${\;r_{\;\text{observed}}\;}_{i}$ from a particular trial i, the mixture model has the following form,

[2] $$\begin{array}{c} r_{i}=\left\{\begin{array}{cc} r(u_{i},l_{i}) & 1-{\;p_{\;\text{swap}}\;}^{i}\;p_{\;\text{misbind}}\;\\ r(l_{i},u_{i}) & {\;p_{\;\text{swap}}\;}^{i}\;p_{\;\text{misbind}}\; \end{array}\right. \end{array}$$

where $\;p_{\;\text{swap}}\;$ is the posterior probability that the given trial has a behavioral swap response (as inferred by the behavioral model, Fig. 1*G*) and $\;p_{\;\text{misbind}}\;$ is a fit parameter (see *The neural mixture model* for more details). Note that the observed, nominal, and misbound responses as well as the probability of a behavioral swap response are all indexed by trial while the probability of a misbinding error $\;p_{\;\text{misbind}}\;$ is not. Intuitively, if $\;p_{\;\text{misbind}}\;=1$, then this means that all of the trials with behavioral swap responses also have neural representations that resemble the misbound representation. If $\;p_{\;\text{misbind}}\;=0$, then this means that all of the trials with behavioral swap responses have neural representations that resemble the nominal representation. We also include a hypothesized explanation for guess trials in the full neural mixture model (*SI Appendix*, *Analysis of guess responses* and Fig. S2).

We do not find strong evidence that behavioral swap errors are associated with misbound neural representations in either monkey (Fig. 2*B*); the aggregate posterior distribution of $\;p_{\;\text{misbind}}\;$ is skewed toward 0 in one monkey (Monkey E: 0.30 to 0.58 probability of alternate rep. on swap trials; Fig. 2*C*, *Top*) and toward 1 in the other (Monkey W: 0.44 to 0.64; Fig. 2*C*, *Bottom*). In both monkeys, however, only a handful of individual sessions have posterior distributions that strongly favor an association between swap errors and misbound representations (Monkey E: significantly greater than 0.25 in 3/13 sessions; Monkey W: 2/10 sessions).

We perform a second, fully cross-validated, sliding window, version of this analysis to confirm these findings across time (Fig. 2*C* and see *Cross–validated neural model* for more details). Here, we used correct trials to fit the linear model described above. Then, for each held-out correct and swap trial, we used the linear model to predict corresponding nominal and misbound representations (Fig. 2*C*, green and purple stars, respectively). These pairs of predicted representations defined a single dimension in population space for each trial, onto which we projected the activity from that held-out trial (Fig. 2*C*, green and purple circles for correct and swap trials, respectively). If swap errors are associated with misbound representations, then we expect swap trials to be closer—on average—to the misbound representation than correct trials are.

The cross-validated analysis finds no difference between correct and swap trials in the second monkey just after the stimuli appear (0 ms to 500 ms after the stimuli appear, Monkey W: activity on swap trials is −0.06 to 0.06 closer to the misbound rep. than on correct trials; Fig. 2*D*, *Bottom Left*), but does find a significant difference prior to the appearance of the cue (−500ms to 0 ms before the cue appears, Monkey W: 0.01 to 0.17; Fig. 2*D*, *Bottom Right*). These results suggest that some swap errors may emerge while multiple stimuli are remembered across a delay period, consistent with some previous theories (15, 16). However, there is no significant difference between swap and correct trials in the first monkey in either time period (0 ms to 500 ms after the stimuli appear, Monkey E: −0.09 to 0.01; −500 ms to 0 ms before the cue appears, Monkey E: −0.03 to 0.08; Fig. 2*D*, *Top* row). Overall, our results suggest that some behavioral swap errors in this task may be due to neural misbinding, but the effect is subject dependent.

### Neural Correlates of Swap Errors Emerge upon Selection from Working Memory.

Next, we perform a similar analysis during the second delay period of the retrospective task. At this point of the task, the monkeys have seen a cue that indicated which of the two items to select and, eventually, report. Therefore, we incorporate a binary “cue” variable, c, into our model:

[3] $$\begin{array}{c} r\left(u,l,c\right)=\left\{\begin{array}{cc} W_{u}^{t}f\left(\;u\;\right)+W_{l}^{d}f\left(\;l\;\right)+b_{1}+\eta & c=1\\ W_{l}^{t}f\left(\;l\;\right)+W_{u}^{d}f\left(\;u\;\right)+b_{0}+\eta & c=0 \end{array}\right., \end{array}$$

where we now have four parameter matrices, one each for the upper and lower color representations when they are the target or distractor, as well as cue-specific offsets $b_{1}$ and $b_{0}$. For example, $W_{u}^{t}$ is the representation of the upper color when it is the target, while $W_{u}^{d}$ is its representation when it is the distractor.

In the second delay period, we hypothesize that behavioral swap responses will be associated with either 1) a misselection of the representation of the target or 2) a misinterpretation of the cue. The misselected color representation has a correct representation of the cue, but the actual target color is represented as the distractor and the actual distractor is represented as the target (Fig. 2 *E*, *Top*, “misselected”). Expressed with our linear model, this would be r(l,u,c). This representation is consistent with an unreliable transformation from the upper-lower color representations in the first delay period to the target-distractor color representations of the second delay period. These upper-lower and target-distractor subspaces have been shown to be orthogonal to each other (22). The misinterpreted cue representation has an incorrect representation of the cue and a representation of the colors that correctly follows the mistaken cue (Fig. 2*E*, *Bottom*, “misinterpreted”). In our model, this is r(u,l,1−c). It is consistent with a misinterpretation of the meaning of the cue that leads to a reliable, but wrong, transformation between the upper-lower and target-distractor color representations. Finally, correct responses were expected to match the nominal representations that faithfully reflect the target and distractor colors, as well as the identity of the cue (Fig. 2*E*, “nominal rep.”).

As before, we incorporated the nominal and two possible swap representations into a mixture model. In both monkeys, the mixture model found strong evidence for neural correlates of swap errors (i.e., trials with a swap error have representations that resemble one of the two alternate representations rather than the nominal representation, Fig. 2*F*; Monkey E: 0.67 to 0.79 probability of alternate rep. on swap trials; Monkey W: 0.74 to 0.88). In addition, almost every individual session also shows strong correlates of swap errors (Monkey E: significantly greater than 0.25 in 12/13 sessions; Monkey W: 9/10 sessions). In one monkey, the misselected color representation is more associated with swap errors than the misinterpreted cue representation (Monkey E: 0.17 to 0.30 greater probability of misselected colors than misinterpreted cue representation; Fig. 2*E*, *Top*). In the second monkey, there is an even split between the two alternate representations (Monkey W: −0.20 to 0.16 greater probability of misselected colors than misinterpreted cue representation; Fig. 2*E*), which may be due to a weaker overall encoding of the cue during the second delay period (0.06 to 0.13 higher cue decoding performance in Monkey E than W).

Next, we adapt the cross-validated projection analysis to the second delay period (Fig. 2 *G*, *Top*). First, we estimate the average, unbiased Euclidean distance between every pair of the three hypothesized representations during the second delay period (see *Characterizing the geometry of error representations* for the details of this analysis). Then, we use these distances to visualize the average representational geometry for these three points within each animal (Fig. 2*G*). This analysis reveals that the distances are, on average, smaller in monkey W than in monkey E (1.34 to 1.92 units longer in monkey E than W). This indicates that the signal to noise ratio of our data for monkey W is less than for monkey E, and may partially be a consequence of the fact that fewer units were recorded (and fewer dimensions retained after preprocessing) for each session on average in monkey W relative to monkey E (on average 18 more dimensions retained after preprocessing in monkey E than W). However, while this would lead to the different representations being less distinguishable from each other overall, we do not believe this fully explains the differences in our results between the two monkeys. This is because the geometry of the representations also differs across monkeys. In particular, the nominal representation is significantly closer to the misselected colors representation than the misinterpreted cue representation in monkey E (Monkey E: 0.09 to 0.95 units further between nominal and misinterpreted cue than nominal and misselected colors), while they are equally distant from each other in monkey W (Monkey W: −0.51 to 0.48 units further). This means that the misinterpreted cue dimension is more orthogonal to the misselected colors dimension in monkey W than in monkey E, and makes the spurious detection of misinterpretation errors less likely.

Next, we apply the projection analysis across time and show that the evidence for a misselected representation on trials with swap errors emerges as the cue is presented in both monkeys (0 ms to 500 ms after the cue comes on, Monkey E: activity on swap trials is 0.08 to 0.21 closer to the misselected rep. than on correct trials; Monkey W: 0.05 to 0.22; Fig. 2 *H*, *Left*). As in the mixture model analysis, we find further evidence for cue interpretation errors in monkey W but not in monkey E by examining the projection of neural activity along the axis connecting the misselected colors and misinterpreted cue representations (Monkey E: activity on swap trials is −0.09 to 0.03 closer to the misinterpreted rep. than on correct trials, along the misinterpreted to misselected axis; Monkey W: 0.05 to 0.26; *SI Appendix*, Fig. S3). This is consistent with monkey E making primarily if not only color selection errors, while monkey W makes color selection errors along with misbinding and cue interpretation errors.

Finally, we compare the strength of the evidence for errors in delay 1 relative to delay 2. In both monkeys, we find stronger evidence for the neural correlates of swap errors in the second delay period (Monkey E: 0.17 to 0.43 greater in delay 2 than delay 1, Monkey W: 0.14 to 0.40, Fig. 2*I*). Overall, our evidence suggests that swap errors primarily occur due to an erroneous transformation between the location-specific (upper or lower) representation and the target-distractor representation. We refer to this as a selection error. We find only inconsistent evidence that swap errors emerge due to an initial misbinding of color to space or a misinterpretation of the meaning of the cue.

### The Prospective Task Also Shows Evidence for Selection Errors.

**Fig. 3.**
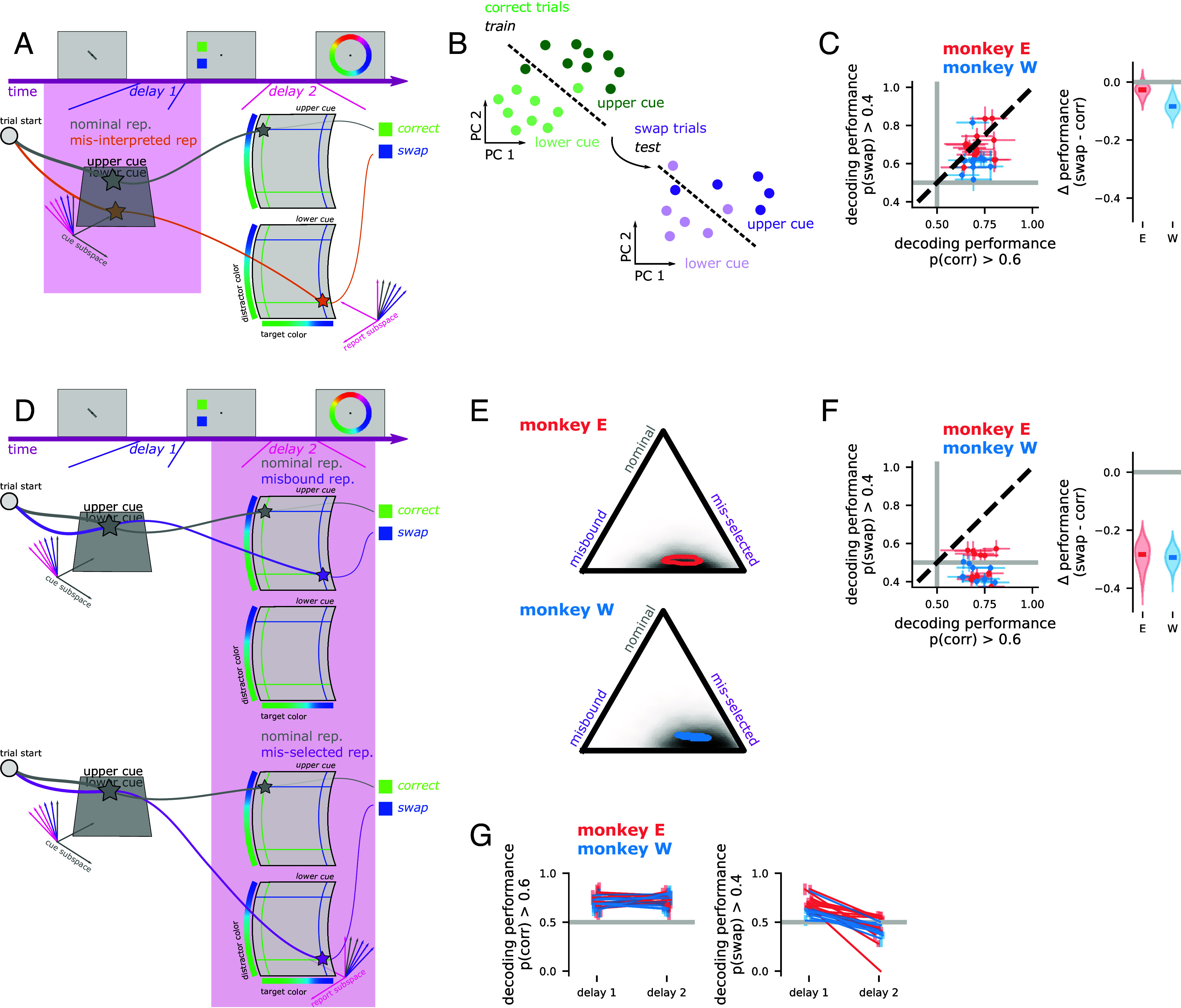
The neural correlates of swap errors in the prospectively cued working memory task. (*A*) Schematic of an example trial in the prospective task, showing the nominal (gray star) and misinterpreted cue (orange star) representations. (*B*) Schematic of the decoder generalization analysis used in this figure. A linear decoder is trained to discriminate between trials with an upper or lower cue using only likely correct trials (*Left*). Then, that same decoder is tested on likely swap trials (*Right*). (*C*) The performance of a decoder trained and tested during delay 1 on likely correct trials (*Left* x-axis) and trained on likely correct but tested on likely swap trials (*Left* y-axis), for each session (points) and the difference between the two averaged across sessions (*Right*). (*D*) Schematic of an example trial in the prospective task, showing the nominal as well as the misbound color (*Top*, purple star) and misselected cue (*Bottom*, pink star) representations. (*E*) The average (contour outline) and aggregate posterior evidence for the association of the nominal, misbound, and misselected representations with swap errors under the neural mixture model. (*F*) The analysis schematized in (*B*) applied to the second delay period. (*G*) The same decoding approach schematized in (*B*) and applied to delay 1 and 2 in (*C* and *F*), respectively. Here, we compare across delay periods for likely correct trials (*Left*) and likely swap trials (*Right*).

Here, we analyzed the neural population activity during the two delay periods of the prospective task. First, we investigated whether behavioral swap errors can be explained as a misinterpretation of the cue during the first delay period (Fig. 3*A*, “misinterpreted rep.”). To do this, we trained a decoder to distinguish between trials with either an upper- or lower-cue, using only trials with likely correct responses. Then, we tested whether or not this decoder successfully generalizes to trials with behavioral swap responses (Fig. 3*B*). If the decoder fails to generalize, then this would indicate that the cue is misremembered or misinterpreted on swap trials (similar to the hypothesis discussed above). In one monkey, we find that the decoder performs just as well on swap trials as on held-out correct trials (Monkey E: −0.08 to 0.01 difference in decoding performance, swap—correct, Fig. 3*C*). In the second monkey, there is a small, but significant decrease in decoder performance between held-out correct trials and swap trials (Monkey W: −0.13 to −0.03, Fig. 3*C*). Overall, we find inconsistent evidence for cue interpretation errors in the first delay period (and see *SI Appendix*, Fig. S7*A* for this analysis over time).

In the second delay period, we can use the same neural mixture model to quantify the likelihood of different alternative representations. This analysis shows strong evidence for neural correlates of swap errors (Monkey E: 0.91 to 0.93 probability of alternate rep. on swap trials; Monkey W: 0.89 to 0.91; Fig. 3*E*). In addition, almost every individual session also shows strong correlates of swap errors (Monkey E: significantly greater than 0.25 in 13/13 sessions; Monkey W: 10/10 sessions). Further, it shows that swap errors in the prospective task are associated with misselected cue representations rather than misbound color representations (Monkey E: 0.12 to 0.36 greater probability of misselected cue than misbound colors representation, Monkey W: 0.27 to 0.50; Fig. 3*E*). Again, we apply the cross-validated version of the analysis across time and show that the misselected cue representation becomes associated with swap errors as the color samples are presented (0 ms to 500 ms after the samples come on, Monkey E: activity on swap trials is 0.16 to 0.24 closer to the misselected rep. than on correct trials; Monkey W: 0.23 to 0.32; *SI Appendix*, Fig. S7*B*). This is consistent with previous work showing the target is selected immediately after stimulus onset (22).

The cue decoding analysis replicates this result. In both monkeys, a decoder trained to infer the cue using only correct trials fails to generalize to swap trials (Monkey E: −0.38 to −0.20 difference in decoding performance, swap—correct, Monkey W: −0.35 to −0.23, Fig. 3*F*). Further, the cue is encoded equally well during the first and second delays on correct trials (Monkey E: −0.03 to 0.05 higher cue decoding performance on correct trials in delay 1 than 2, Monkey W: −0.06 to 0.02; Fig. 3*G*, *Left*); the decrease in cue decoding only emerges on behavioral swap trials (Monkey E: 0.17 to 0.35 higher cue decoding performance on swap trials in delay 1 than 2, Monkey W: 0.13 to 0.24; Fig. 3 G, *Right*). Overall, in the prospective task, we find evidence for what we term cue selection errors—where the cue is remembered correctly over the first delay period, but is corrupted when it is retrieved from working memory and used to interpret the incoming color information.

## Discussion

Our results provide insight into the neural mechanisms underlying “swap” errors. In both monkeys, we find evidence that errors are introduced during the selection of information from working memory. In one monkey, we found results that were consistent with the animal misbinding color and misinterpreting cue representations. This suggests misbinding errors and cue-related confusion may still explain some behavioral swap errors in some individuals. Furthermore, neither monkey showed evidence that swap errors could be explained by failing to encode or by forgetting one of the two stimuli after they were presented (*SI Appendix*, *Stimulus forgetting* and Fig. S4).

Extant theories of swap errors have not generally considered “selection errors” as a major factor. Instead, they have tended to focus on errors in storage or encoding, whether capacity limits (20, 27, 28), feature misbinding (15, 16), or misinterpretation of the cue (20, 21). These explanations are focused on imperfections in the representations of remembered stimuli, which then lead to an incorrect stimulus being selected for report. Alternatively, the presence of selection errors raises the possibility that errors are also—or even primarily—due to errors in how working memory is used. By analogy, if one writes a shopping list down correctly but still buys the wrong items, the fault does not lie with the shopping list—it lies with how the shopper interacted with the list. In our case, we find a largely correct “shopping list” in the first delay period, yet the wrong item is still selected. Thus, we argue that many swap errors emerge due to errors in the cognitive processes that interact with working memory, or with the interaction itself. To our knowledge, most theoretical work aimed at understanding errors in behavioral tasks in neuroscience has focused on ways in which those errors can emerge from the underlying representations of specific task-relevant stimuli (15, 16, 18, 29–31). Our work demonstrates an alternative—that many errors emerge instead from erroneous selection or control of representations.

Recent work used functional MRI to investigate the neural basis of swap errors (21). This work showed that, on swap trials, a representation of the to-be-reported position emerged prior to the response period—and that this representation had a similar timecourse to the timecourse on correct trials. Our results are consistent with this finding and extend it in two important ways. In particular, this prior work did not yield insight into the neural representations associated with swap errors prior to the cue nor did it analyze the representation of the cue itself. Thus, it left open the possibility that swap errors emerge due to misbinding of stimulus features either during stimulus presentation or during a memory delay as well as swap errors that emerge due to a misinterpretation of the cue. Our work finds evidence for both of these forms of swap errors in one animal. However, the balance of evidence in our study supports the idea that swap errors primarily emerge during the selection process itself—and are not necessarily anticipated by erroneous representations during the preceding memory delay. This pattern of results replicates in a second, prospectively cued task where the neural basis of swap errors had not been studied previously.

One potential explanation for swap errors is that they represent an educated guess, where participants report a random, but recently seen stimulus when they do not know how else to respond (20). While our work and previous work (21) indicate that the participant has made up their mind about how to respond at least by the second delay period, swap errors could still result from an educated guess at the time of the cue presentation. This idea is quite similar to our idea of a selection error, but would differ in the expected confidence of the subject in their response. For an educated guess, we expect that the subject would be less confident in their eventual response—while in the selection error case, we expect the subject to be equally as confident as when they are correct. In humans, many swap errors are associated with high confidence (21). However, we do not have an explicit readout of confidence from our subjects, and this leaves open the possibility that there is a difference in confidence between correct and swap responses in our case. One proxy for decision confidence in animals is the response time (32), where less confident decisions are made more slowly than more confident decisions. Here, we find no difference in average response time between correct and swap trials in one monkey (*SI Appendix*, Figs. S5 and S6*A*; Monkey E, retrospective trials: correct—swap median difference =−3 ms to 3 ms; Monkey E, prospective trials: correct—swap median difference =−4 ms to 2 ms), but a small difference is present in the other monkey (*SI Appendix*, Figs. S5 and S6*D*; Monkey W, retrospective trials: correct—swap median difference =−20 ms to −4 ms; Monkey W, prospective trials: correct—swap median difference =−12 ms to −3 ms). Excluding all trials with long response times does not affect our results in either monkey for the retrospective (*SI Appendix*, Fig. S5) or prospective tasks (*SI Appendix*, Fig. S6; see *SI Appendix*, *Reaction time analyses* for more detail). Thus, this difference in reaction time present in one monkey does not drive either our main results or the differences we observe between animals. Overall, these results argue against the idea that swap errors in this context are educated guesses.

How do these selection errors actually occur in the brain? Our work and related theoretical work on working memory tasks like this one leave this question open. Interestingly, recurrent neural networks trained to perform this (33) and similar (34) tasks do produce some swap errors, but at much lower rates than in our data and comparable data from humans (4, 11, 16, 21). Thus, while these models replicate the neural population dynamics on correct trials (33), they have so far yielded little insight into the mechanisms of swap errors. One possibility is that recurrent neural networks trained to perform a single working memory task are able to specialize to the specific conditions of the task, while the networks in the brain that perform such tasks must remain flexible. For instance, in our case, a remembered color stimulus must be selected from one of two position-based subspaces on the basis of a binary cue. In general, however, humans and other animals must be able to remember stimuli presented at any position and select from among these memories based on a variety of potential cues. Such flexibility could interact with capacity limitations to make selection errors more likely—by, for example, making the gain modulation or subspace rotation control process used for stimulus selection vulnerable to noise. The noise patterns that produce errors in the control process then need not be aligned with the representations of the remembered stimuli or of the cue.

Overall, this work deepens our understanding of the neural basis of swap errors and highlights the importance of selection from working memory as a source of errors more generally. Further work is necessary to understand how these findings generalize to other variants of working memory recall tasks as well as to novel tasks. One striking finding from the working memory literature is that swap errors increase in frequency for stimuli that are presented at nearby positions in visual space (16). This can easily be explained through a representation error framework (16), but does not have a clear explanation under a selection error framework. Thus, for nearby stimuli, it is possible that representational swap errors become more common, but that selection errors produce a baseline rate of swap errors for stimuli at any distance. Further work can test this intuition and further tease these two putative sources of swap errors further apart.

## Materials and Methods

### Subjects.

The experiments were performed in two adult Rhesus macaques (Macaca mulatta; 8 to 9 y old). Monkey E and Monkey W weighed 12.1 and 8.9 kg, respectively. All experimental procedures were approved by the Princeton University Institutional Animal Care and Use Committee and were in accordance with the policies and procedures of the NIH.

### Behavioral Tasks.

The behavioral task was implemented in Psychtoolbox and MATLAB (Mathworks) and it was displayed on a Dell U2413 liquid crystal display (LCD) monitor, which the monkeys viewed at a distance of 58 cm. During the task, the animals were required to remember the two square stimuli presented at 45° clockwise (upper) and anticlockwise (lower) from the horizontal meridian and with an eccentricity of 5 degrees of visual angle. The colors were sampled from 64 uniformly spaced points on a photometrically isoluminant circle in CIELAB color space, centered at (L=60, a=6, and b=14) with a radius of 57 units. The two colors for each trial were sampled independently.

During an experimental session, the animal performed two different tasks in a blocked fashion. The first task is referred to as the retrospective task (retro). This tasks begins with a fixation period (500 ms) before two colored squares appeared on the screen for 500 ms. Then, after a delay period (500 ms or 1,000 ms), a cue was presented in the fixation window (and subtending 2°) for 300 ms, which indicated to the animal which of the two colors should be reported. In the experiments, two distinct sets of cues were used: 1) lines oriented 45° clockwise or anticlockwise from the horizontal meridian indicating the lower or upper position, respectively; and 2) a triangle or a circle, indicating the lower or upper position respectively. Next, after a second delay period (500 ms to 700 ms), a 2° wide color wheel centered on the fixation point appeared and the animal signaled their chosen color with a saccade to the corresponding location on the color wheel. The color wheel was randomly oriented on each trial to prevent motor planning or encoding of a spatial memory prior to the presentation of the wheel. The animals were rewarded according to the distance of their response from the target color (maximum of twelve drops, precise reward determined by a von Mises distribution centered at 0° error with a SD of 22°). They were not rewarded if their response was greater than 40° or 60° from the target, for Monkey E and W respectively.

The second task is referred to as the prospective task (pro). In this task, after a fixation period (500 ms), the cue was shown (300 ms), followed by a delay period (200 ms to 600 ms). Then, the two colored squares were shown (500 ms) and after a second delay period (Monkey E: 1,000 ms to 2,000 ms; Monkey W: 1,300 ms to 2,000 ms), the animal made their response through a saccade onto the color wheel. The details of the color wheel and reward scheme were the same as for the retrospective task.

The two tasks were performed in randomly interleaved blocks. There were three blocks: prospective trials with the first cue set, retrospective trials with the first cue set, and retrospective trials with the second cue set. Block transitions occurred after 60 correct trials on the prospective task or 30 correct trials on either of the retrospective blocks (thus balancing the number of prospective and retrospective trials).

Throughout the whole experimental session, the eye position of the animal was monitored at 1 kHz through video-based eye tracker (SR Research). The monkeys had to maintain their fixation within 2° of a central fixation cross until the response period after the second delay. The trial was aborted and the monkey received a short time out if they moved their eye out of this window.

In addition to the two main experimental conditions that we analyzed here, the monkeys also both performed a version of the task where only a single color was shown. We do not show any primary analyses of these data, though we use it in the fitting of our neural mixture model (*The neural mixture model*).

Monkey E completed 11,131 trials over 13 sessions and Monkey W completed 9,865 trials over 10 sessions.

### Electrophysiological Recordings.

To immobilize the head, monkeys were implanted with a titanium headpost. Both monkeys were also implanted with two titanium recording chambers, which were positioned using 3D models of the skull to allow for recordings from lateral prefrontal cortex, frontal eye fields partial cortex, and V4.

Neural activity was recorded via up to 80 simultaneously inserted epoxy-coated tungsten electrodes (FHC). The electrodes were allowed to settle for 2 to 3 h before recordings. Broadband (30 kHz) activity was recorded from each electrode (Blackrock Microsystems).

### The Behavioral Model.

To classify responses as correct, a swap, or a guess, we modeled the responses on each trial as a mixture of three different possible response types: a correct response, swap response, and guess response. In particular, we fit the model:

[4] $$\begin{array}{cc} p^{i}\left(\;c_{\;\text{report}}\;\right) & ={\;p_{\;\text{correct}}\;}^{i}N\left(\;c_{\;\text{report}}\;-\;t\;,\sigma^{i}\right)\\ & \;+{\;p_{\;\text{swap}}\;}^{i}N\left(\;c_{\;\text{report}}\;-\;d\;,\sigma^{i}\right)+{\;p_{\;\text{guess}}\;}^{i}\frac{1}{2\pi }, \end{array}$$

where ${\;p_{\;\text{correct}}\;}^{i}=1-{\;p_{\;\text{swap}}\;}^{i}-{\;p_{\;\text{guess}}\;}^{i}$ and N(μ,σ) is the probability distribution function of a normal distribution with mean μ and SD σ. The index i related to experimental sessions. We fit the model in a hierarchical manner within each monkey, where the probability parameters were modeled as arising from a distribution across distinct sessions. In particular, we reformulated the problem using the transformation:

[5] $$\begin{array}{c} {\;p_{\;\text{guess}}\;}^{i}=\frac{\exp\left[w_{\;\text{guess}}^{i}\right]}{\exp\left[w_{\;\text{guess}}^{i}\right]+\exp\left[w_{\;\text{swap}}^{i}\right]+1} \end{array}$$

and similarly for ${\;p_{\;\text{swap}}\;}^{i}$ while ${\;p_{\;\text{correct}}\;}^{i}=1-{\;p_{\;\text{guess}}\;}^{i}-{\;p_{\;\text{swap}}\;}^{i}$. Then, we modeled $w_{\;\text{type}}^{i}∼N\left(w_{\;\text{type}},s_{\;\text{type}}\right)$ for guesses and swaps. This hierarchical approach provides the benefit of partial pooling, where the different error rates on each individual session are not assumed to be exactly the same nor are they assumed to be completely independent (35). In total, our behavioral model had three parameters for each session ($\;p_{\;\text{swap}}\;$, $\;p_{\;\text{guess}}\;$, σ) and four parameters at the level of each monkey ($w_{\;\text{swap}}$, $w_{\;\text{guess}}$, $s_{\;\text{swap}}$, $s_{\;\text{guess}}$. The monkey-level mean parameters were given normal priors with mean 0 and SD 1, while the SD parameters were all given half-normal priors with mean 1 SD 3. We obtained qualitatively similar results when fitting the models at the per session level with a uniform Dirichlet prior for the probability parameters.

We also implemented an inverse model from which we could sample from a posterior predictive distribution of response errors. In particular,

[6] $$\begin{array}{c} E\left(\;t\;,\;d\;\right)∼\left\{\begin{array}{cc} N(0,\sigma ) & p=\;p_{\;\text{correct}}\;\\ N(\;d\;-\;t\;,\sigma ) & p=\;p_{\;\text{swap}}\;\\ U(-\pi ,\pi ) & p=\;p_{\;\text{guess}}\; \end{array}\right. \end{array}$$

where all values are treated as periodic between −π and π.

Finally, for each trial, we also computed the posterior probability that the response arose from each of the three categories that we considered, according to Bayes rule:

[7] $$\begin{array}{cc} p(\;\text{type}|r) & =\frac{p(\;\text{type})p(r|\;\text{type})}{p(r)} \end{array}$$

#### Fitting procedure.

The models were fit using Hamiltonian Monte Carlo (HMC) (36) with No-U-Turn Sampling (37) implemented in the probabilistic programming language Stan (38) which we interacted with via the pystan (39) package. For each model, we ran four chains and for 500 warmup iterations and 500 samples. We verified that the model had converged via the $\hat{r}$ scores for each parameter, which quantifies how well the different chains have converged to the same distribution. In particular, $\hat{r}$ is 1 if the chains have converged to the same distribution and >1 otherwise. Each parameter from the fit models reported here had $\hat{r}=1$. We also evaluated other metrics of fit, which yielded no warnings.

### Hypothesized Error Types.

Across our two primary epochs of interest, we attempt to explain swap errors (that is, the behavioral swap response) in four different ways.

#### Misbinding errors.

In this case, either when the stimuli are initially encoded or while they are being stored in working memory over the first delay period, the color and location associations are misbound to each other—such that a green color in the upper location and a blue color in the lower location are represented as if they were a blue color in the upper location and green color in the lower location. We incorporated this possibility into our mixture model as described below (*The mixture model*). This form of error has been theorized for decades, both in psychology (14–16) and in neuroscience (17, 18, 40).

#### Cue interpretation errors.

In this case, the meaning of the cue is misinterpreted on certain trials (e.g., a cue indicating the animal should report the color at the lower position is interpreted to mean that the animal should report the color at the upper position), which leads to a behavioral swap response. We hypothesize that this difference would manifest in the neural representation of the cue. In particular, we expect that the neural representation would reflect the animal’s belief about what the cue was—and on swap trials, this would look like a representation of the incorrect cue.

#### Selection errors.

In both tasks, the animal first remembers one piece of information and then has to combine that piece of information with a second piece of information. In particular, in the retrospective task, the animal first sees two colors and must remember both the colors and their positions. Then, the animal is shown a cue. To solve the task, the animal has to use that cue to select one of the two remembered colors (based on its position) for eventual report. For selection errors, we hypothesize that it is this selection of information that was previously (and successfully) stored in working memory that gives rise to swap errors, by leading to an error in encoding when it is selected.

#### Forgetting.

One potential explanation for swap errors is that, just after the stimuli have been presented, the animal simply forgets one of the two stimuli. Then, when presented with the response wheel, the animal simply reports the color that they remember even though they know that it may not be correct. We had difficulty incorporating this forgetting possibility into our mixture model, so we instead worked to evaluate the number of stimuli encoded during the second delay period (either 1 or 2), using a subset of trials on which a single stimulus was presented. Here, we show that swap errors are not associated with representations that look like those on trials where a single stimulus had been presented (for more detail see *SI Appendix*, *Stimulus forgetting*), which is inconsistent with the educated guessing explanation for swap errors proposed in ref. 20.

### Electrophysiological Data Preprocessing and Exclusion.

We include both single and multiunits in our analyses. We exclude units that have zero firing rate for a contiguous 50 trial block in the session from analysis. After this exclusion, we bin the firing rates into 500 ms bins (except in the case of the first delay period on prospective trials, since the delay period is only 200 ms, we use only 200 ms bins) and then z-score each neuron individually before performing principal components analysis and retaining 95% of the variance. In the cross-validated analyses, both of these transformations are fit to only training-set trials, then applied to the test-set trials.

### The Neural Mixture Model.

To model the neural data, we also take a mixture modeling approach. The model can be understood as having two parts. The first part models the responses to the two presented colors and (in delay 2) to the cue. The second part of the model models the dependence of the responses on the eventual behavioral response—i.e., a correct response, swap error, or guess. We will describe these two components in sequence.

#### Additional preprocessing.

For this model, we add two additional preprocessing steps, which do not qualitatively affect our conclusions but which do improve the convergence times of our models. Even after our exclusion of neurons with consistently zero firing rates for 50 trials in a row during the session, many neurons in the population have zero firing rate for some contiguous portion of the session. To ameliorate this, we impute the firing rate of those neurons for their stretch of contiguously zero firing based on the five nearest neighbor trials across the population [KNNImputer in sklearn (41)].

We also z-score the firing of each unit after the principal components analysis (PCA) transform, since we use a uniform prior variance for the firing of each unit in the statistical model.

#### The color and cue representations.

We model the color representations using two sets of periodic splines. In particular, for a particular color c∈[0,2π], we map it into a spline representation with the function f(·), which moves from a single number to a K-dimensional representation where K is the number of “knots” in the spline representation. We also control the smoothness of the splines with an order parameter. We performed all of our analyses with K= 4, 5, and 6 and order =1,2. The results did not qualitatively change for any of these choices. We report results from models with splines chosen to have K=5 and order =1.

Once we have the spline representation of both the upper and lower (target and distractor) colors in delay 1 (delay 2), then we model the neural responses r as a linear model of both colors:

[8] $$\begin{array}{cc} r(\;u\;,\;l\;) & =W_{u}f\left(\;u\;\right)+W_{l}f\left(\;l\;\right)+\eta , \end{array}$$

where $W_{u}$ and $W_{l}$ are fit parameters, u and l are the upper and lower colors (for delay 1), and η∼N(0,σ). Thus, the parameters fit for this part of the model are $W_{u}$ and $W_{l}$, which are each N×K matrices and σ, which is a N×1 vector (we do not fit a full covariance matrix), where N is the number of neurons (or the number of dimensions after PCA) and K is again the number of knots in the spline basis.

To model the cue, we simply add a binary variable c which indicates whether the upper color (c=1) or lower color (c=0) are cued for report as well as cue-specific intercept terms $b_{0}$ and $b_{1}$:

[9] $$\begin{array}{c} r\left(u,l,c\right)=\left\{\begin{array}{cc} W_{u}^{t}f\left(\;u\;\right)+W_{l}^{d}f\left(\;l\;\right)+b_{0}+\eta & c=1\\ W_{l}^{t}f\left(\;l\;\right)+W_{u}^{d}f\left(\;u\;\right)+b_{1}+\eta & c=0 \end{array}\right. \end{array}$$

Now there are four matrix parameters, for the upper and lower colors when they are target or distractor. For example, $W_{u}^{t}$ is the representation of the upper color when it is the target, while $W_{u}^{d}$ is its representation when it is the distractor. This allows for a flexible arrangement of the color representations.

#### The mixture model.

We include a mixture component with the model described above, where for a given trial during delay 1, we fit,

[10] $$\begin{array}{cc} P({\text{r}}_{\text{​}\text{observed}}|\text{u},l)) & =p_{C}P\left({\text{r}}_{\text{​}\text{observed}}|\bar{r}(\text{u},l),\sigma \right) \end{array}$$

[11] $$\begin{array}{cc} \;\;\;\;\;\;\;\;\;\;\;\;\;\;\;\;\;\;\;\;\;\;\;\;\;\;\;\;\;\;\;\; & +{\text{p}}_{\text{​}\text{swap}}\;{\text{p}}_{\text{​}\text{misbind}}\;P\left({\text{r}}_{\text{​}\text{observed}}|\bar{r}(l,\text{u}),\sigma \right) \end{array}$$

[12] $$\begin{array}{cc} \;\;\;\;\;\;\;\;\;\;\;\;\;\;\;\;\;\;\;\;\;\;\;\;\;\;\;\;\;\;\;\; & +{\text{p}}_{\text{​}\text{guess}}\;{\text{p}}_{\text{​}\text{resp}}\;p\left({\text{r}}_{\text{​}\text{observed}}|\bar{r}({\text{c}}_{\text{​}\text{report}},l),\sigma \right) \end{array}$$

where $p_{C}=\;p_{\;\text{correct}}\;+\;p_{\;\text{swap}}\;\left(1-\;p_{\;\text{misbind}}\;\right)+\;p_{\;\text{guess}}\;\left(1-\;p_{\;\text{resp}}\;\right)$ is the accumulated probability of the correct representation, $\;p_{\;\text{correct}},\;p_{\;\text{swap}},\;p_{\;\text{guess}}\;$ are taken from the behavioral model and are not fit, $\;p_{\;\text{misbind}}\;$ and $\;p_{\;\text{resp}}\;$ are free scalar parameters that represent the likelihood that a given response looks like either a misbinding error (u and l are swapped) or a particular version of a guess, in which the representation of the eventually reported color looks more like what the animal eventually reports ($\;c_{\;\text{report}}\;$) than the actual color (as in this example, u). This part of the full model adds two parameters to the total set of parameters fit for the neural mixture model. Importantly, all of the parameters are fit simultaneously, rather than in two stages.

The model for delay 2 in the retrospective task is similar but has another possibility for swap errors that relates to the encoding of the cue. Here, we take an example trial where the cue indicates that the upper stimulus is the target,

[13] $$\begin{array}{cc} P({\text{r}}_{\text{​}\text{observed}}(\text{t},\text{d},\text{​}\text{upper})) & =p_{C}P\left({\text{r}}_{\text{​}\text{observed}}|\bar{r}(\text{u},l,1),\sigma \right) \end{array}$$

[14] $$\begin{array}{cc} \;\;\;\;\;\;\;\;\;\;\;\;\;\;\;\;\;\;\;\;\;\;\;\;\;\;\;\;\;\;\;\;\;\;\;\;\;\;\;\;\;\;\;\;\;\; & +{\text{p}}_{\text{​}\text{swap}}\;{\text{p}}_{\text{​}\text{selection}}\;P\left({\text{r}}_{\text{​}\text{observed}}|\bar{r}(l,\text{u},1),\sigma \right) \end{array}$$

[15] $$\begin{array}{cc} \;\;\;\;\;\;\;\;\;\;\;\;\;\;\;\;\;\;\;\;\;\;\;\;\;\;\;\;\;\;\;\;\;\;\;\;\;\;\;\; & \;+{\text{p}}_{\text{​}\text{swap}}\;{\text{p}}_{\text{​}\text{cue}}\;P\left({\text{r}}_{\text{​}\text{observed}}|\bar{r}(\text{u},l,0),\sigma \right) \end{array}$$

[16] $$\begin{array}{cc} \;\;\;\;\;\;\;\;\;\;\;\;\;\;\;\;\;\;\;\;\;\;\;\;\;\;\;\;\;\;\;\;\;\;\;\;\;\;\;\; & \;+{\text{p}}_{\text{​}\text{guess}}\;{\text{p}}_{\text{​}\text{resp}}\;p\left({\text{r}}_{\text{​}\text{observed}}|\bar{r}({\text{c}}_{\text{​}\text{report}},l,1),\sigma \right) \end{array}$$

where $p_{C}=\;p_{\;\text{correct}}\;+\;p_{\;\text{swap}}\;\left(1-\;p_{\;\text{selection}}\;-\;p_{\;\text{cue}}\;\right)+\;p_{\;\text{guess}}\;\left(1-\;p_{\;\text{resp}}\;\right)$ is the accumulated probability of the correct representation.

The model for delay 2 in the prospective task is identical, but the interpretation of the mixture parameters is different. In particular, $\;p_{\;\text{selection}}\;$ from the retrospective task is analogous to $\;p_{\;\text{misbind}}\;$ and $\;p_{\;\text{cue}}\;$ is analogous to $\;p_{\;\text{selection}}\;$, as discussed in the main text.

#### Hierarchical structure for the delay 2 models.

To improve the quality of our parameter estimates, we adopt a hierarchical structure for our delay 2 model, since the set of predictions that we test are the same across both the retrospective and prospective tasks. In this structure, we fit

[17] $$\begin{array}{c} {\overset{¯}{r}}_{2}^{\;\text{type}}\left(\;d\;,\;t\;,\;\text{upper}\right) \end{array}$$

for each type∈{retro,pro,single} and where each of the type-specific W and z are assumed to be drawn from a normal distribution with a mean and SD that is fit across all the types. These combined mean and SD parameters are given normal and half-normal priors, respectively—both with SD 10. As with the behavioral model, this allows us to partially pool information across the different task conditions, where the representation of color is not assumed to be exactly the same across tasks—but nor is it assumed to be completely independent.

#### Fitting procedure.

As for the behavioral model, we fit this model using HMC in Stan. For each model, we ran four chains and for 500 warmup iterations and 500 samples. We verified that the model had converged via the $\hat{r}$ scores for each parameter, which quantifies how well the different chains have converged to the same distribution. In particular, $\hat{r}$ is 1 if the chains have converged to the same distribution and >1 otherwise. Each parameter from the fit models reported here had $\hat{r}\approx 1$. We also evaluated other metrics of fit, which yielded no warnings.

### Cue Decoding Analysis.

For the cue decoding analysis, we began by splitting the trials into likely correct trials ($\;p_{\;\text{correct}}\;>0.6$) and likely swap trials ($\;p_{\;\text{swap}}\;>0.4$). Then, we train a linear classifier to decode the value of the cue on the likely correct trials. The reported decoding performance is the cross-validated performance of the decoder on those trials. Next, we evaluate the performance of the same decoder to all of the likely swap trials.

Thus, if likely correct and likely swap trials have similar representations of the cue, we expect the decoder to generalize (as for the first delay period of the prospective task). However, if the representations differ, then the decoder will not generalize (as for the second delay period of the prospective task).

### Cross-Validated Neural Model.

To evaluate how the observed effects change over time as well as to replicate our findings from the Bayesian neural mixture model with more familiar techniques, we develop a fully cross-validated version of the mixture model analysis. Here, we again divide the trials into likely correct ($\;p_{\;\text{correct}}\;>0.3$) and likely swap ($\;p_{\;\text{swap}}\;>0.3$) trials. We also exclude any trials where the target and distractor colors are closer than $\frac{\pi }{4}$ radians apart. Then, we fit the model described in section *The color and cue representations* as a standard Ridge regression using only likely correct trials in a leave-one-out scheme. Thus, for that particular fit, we have one likely correct trial that has not been used to fit the model as well as several likely swap trials. For each of those trials, we use the model to construct our usual hypothesis space (e.g., the prototypical correct and spatially misbound representations from the first delay period of the retrospective task) and we evaluate the projection of the held-out trials on the dimension connecting these different prototypes. In particular, for each held out trial r and two prototypes ${\overset{¯}{r}}_{A}$ and ${\overset{¯}{r}}_{B}$, we compute,

[18] $$\begin{array}{cc} x & =\frac{{\overset{¯}{r}}_{A}-{\overset{¯}{r}}_{B}}{\left|\right|{\overset{¯}{r}}_{A}-{\overset{¯}{r}}_{B}{\left|\right|}_{2}^{2}}\cdot r \end{array}$$

and x will be close to zero if r is close to $r_{B}$ while it will be close to one if r is close to $r_{A}$. Thus, to evaluate the evidence for swap errors, we compare the average value of x taken across likely correct trials with the average value taken across likely swap trials.

In this analysis, we consider all of the same prototypes that we consider in the mixture model.

### Characterizing the Geometry of Error Representations.

To visualize the geometry of these error representations and to aid in interpreting the projection analysis (Fig. 2 and *SI Appendix*, Figs. S3 and S7), we compute the average, cross-validated Euclidean distance between each of the three hypothesized representations during the second delay period. The cross-validation allows us to avoid the positive bias common to most distance estimations in noisy data (42); so, here, zero distance means that the average activity in two conditions is indistinguishable. The representations are estimated only from likely correct trials. To proceed, we find all pairs of trials where one trial is similar to one representation (e.g., the nominal representation) and the other trial is similar to one of the other hypothesized representations (e.g., the misselected cue representation). We compute the vector connecting these two points, $t_{1}-$$t_{2}=v_{A}$. Then, we take all trials similar to this pair of trials (but excluding the pair itself) and estimate the vector connecting them, ${\overset{¯}{t}}_{1}-{\overset{¯}{t}}_{2}=v_{B}$. The dot product of these two vectors represents an unbiased estimate of the Euclidean distance between the representations of the first and second condition, $d_{12}=v_{A}\cdot v_{B}$ (42). We repeat this procedure for all valid pairs of trials and all pairs of hypothesized representations.

Thus, we obtain a 3×3 distance matrix between the three hypothesized representations (retro: nominal, misselected colors, misinterpreted cue; pro: nominal, misbound, misselected cue) corresponding to each session. Then, we average these matrices across sessions to obtain a single average distance matrix for each monkey. Finally, we use multidimensional scaling to embed these three points into two-dimensional space. Because there are only three points, they are embedded into a two-dimensional space without any distortion.

## Supplementary Material

Appendix 01 (PDF)

## Data Availability

The specific version of the code underlying the analyses in this paper is available online through Zenodo (43). The data underlying the figures are available on Figshare (44).
