# Supplementary material for: The neural basis of swap errors in working memory
Source: Proc Natl Acad Sci U S A. 2024 Aug 5;121(33):e2401032121. doi: 10.1073/pnas.2401032121 (PMC11331092; doi:10.1073/pnas.2401032121)
Supplement: Supplementary file 1 — Appendix 01 (PDF) [file pnas.2401032121.sapp.pdf]

S1 Supplement

S1 Region-dropping analysis

To understand whether the different regions recorded here contribute differently to the neural correlates of swap errors, we have performed a region dropping analysis using the cross-validated timecourse model described in the main text. To evaluate the contribution of a particular region, we fit the model as described in the main text and calculate the difference between correct and swap traces. Then, we evaluate how this difference changes, when we exclude either all of the neurons from a single region or an equivalently sized set of random neurons. We choose 100 random, equally sized subsets to remove. If a particular region is more important for the neural correlates of swap errors than average, then this difference will be positive (in the figure, we refer to this as the relative importance). If a region is less important than average, then this difference in effects will be negative.

Overall, this analysis finds only inconsistent regional effects (fig. S1). There is some evidence in Monkey W and specifically in the prospective task (fig. S1, right) that the neural correlates of swap errors emerge first in V4/PIT (i.e., evidence for swap errors during delay 1) and then transition into PFC (i.e., evidence for swap errors during delay 2) – but the second monkey does not show a reliable effect in the same direction. This analysis supports the idea that working memory representations are broadly distributed across different brain regions.

S2 Analysis of guess responses

The neural mixture model finds high probability that guess trials resemble representations of the eventually reported stimulus in both the first (fig. S2b) and second (fig. S2c delay periods of the retrospective task. However, the cue identity appears to be reliably encoded in the second delay period, indicating that the animal does not forget all experimental variables on guess trials (fig. S2d). The same pattern of results holds in the prospective task (fig. S2e, f, g), though there is a less reliable representation on the cue on likely guess trials in one of the two monkeys.

S3 Stimulus forgetting

We investigate whether the the neural activity on swap trials is consistent with a representation of just one rather than two total stimuli. This addresses the explanation for swap errors proposed in [1].

To do this, we train a linear decoder to discriminate between trials from the retrospective (fig. S4a) or prospective (fig. S4b) tasks (both in the second delay period) and trials on which only a single color was shown. Then, we test evaluate the generalization performance of that decoder on likely swap trials. If behavioral swap errors result from only a single stimulus being remembered and the animal simply reporting that remembered stimulus, then we expect that the decoder will fail to generalize. However, we find that the decoder performs just as well on swap trials as on correct trials.

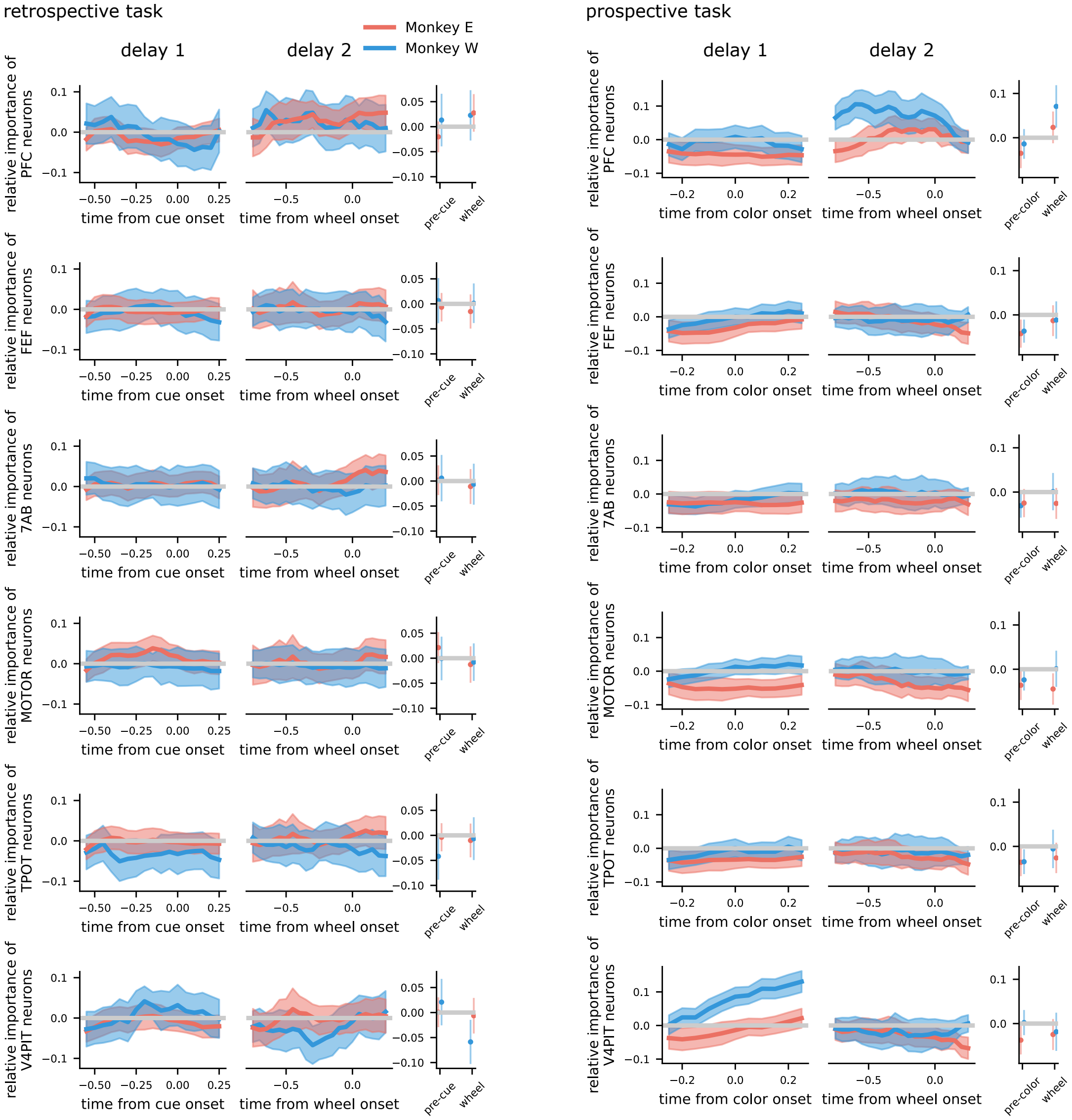

Figure S1: The unique contribution of different brain regions to the neural correlates of swap errors. (left) The region dropping analysis applied to delay 1 and delay 2 of the retrospective task. The right column shows the relative important at the specific times reported in the main text. There are not consistent effects across monkeys. (right) The region dropping analysis applied to delay 1 and delay 2 of the prospective task. The layout is the same as for the retrospective task and there are no consistent effects across monkeys.

S4 Reaction time analyses

Both subjects have similar reaction times for all response types in both the retrospective (Monkey E, retrospective trials: correct trial median RT = 244 ms to 247 ms; swap trial median RT = 242 ms to 248 ms; guess trial median RT = 244 ms to 250 ms ; Monkey W, retrospective trials: correct trial median RT = 273 ms to 275 ms; swap trial median RT = 278 ms to 295 ms; guess trial median RT = 285 ms to 298 ms) and prospective tasks (Monkey E, prospective trials: correct trial median RT = 245 ms to 247 ms; swap trial median RT = 244 ms to 250 ms; guess trial median RT = 240 ms to 250 ms ; Monkey W, prospective trials: correct trial median RT = 275 ms to 278 ms; swap trial median RT = 280 ms to 288 ms; guess trial median RT = 270 ms to 293 ms). In monkey W, there is a bimodal distribution of reaction times (figs. S5 and S6d). One possibility is that this bimodality reflects two distinct processes, potentially confident and non-confident responses, and

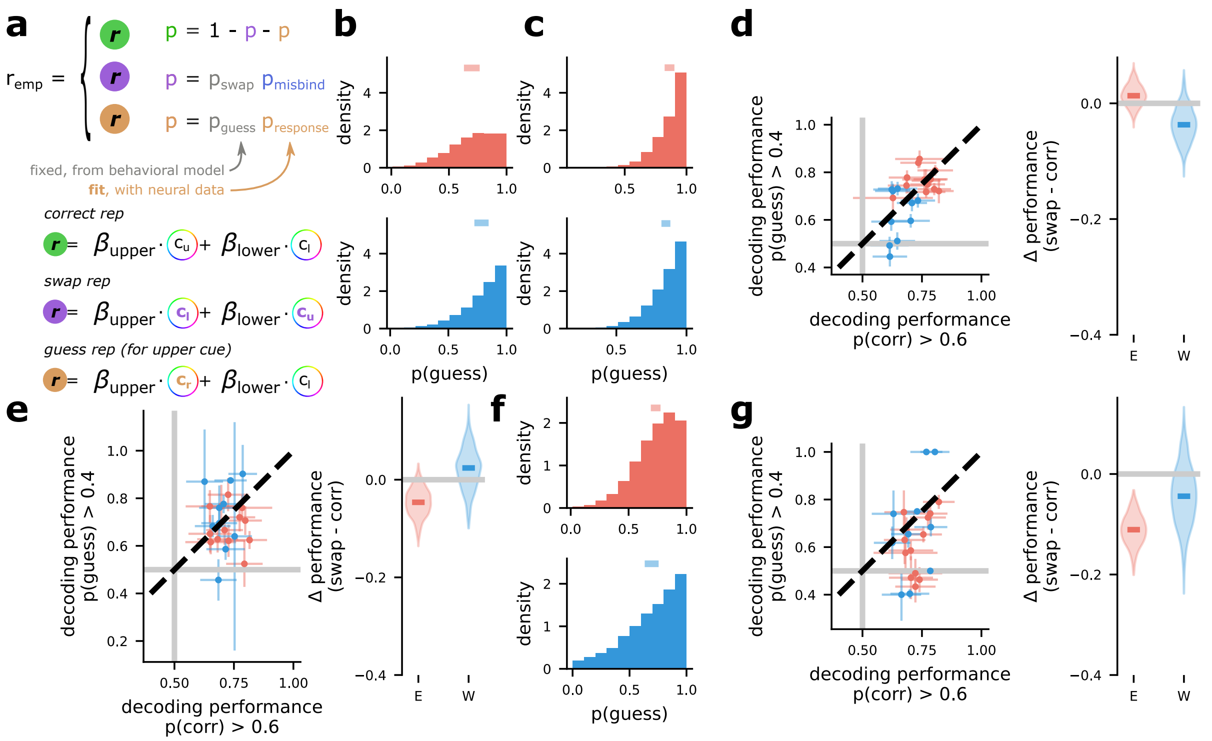

Figure S2: The neural correlates of guess responses in retrospective and prospective working memory tasks. **a** Schematic of the mixture modeling approach used to express guess responses. Here, we assume that the neural activity on guess trials will represent the eventually reported color (c_r), even when that color is neither the target nor the distractor. **b** Average (line) and aggregate posterior (distribution) across sessions for the p_{response} parameter shown in **a** during delay 1 of the retrospective task. **c** The same as **b** but for delay 2 of the retrospective task. **d** The decoder generalization analysis applied to delay 2 of the retrospective task, but instead of contrasting likely correct and likely swap trials the analysis contrasts likely correct and likely guess trials. **e** The same as **d** but applied to delay 1 of the prospective task. **f** The same as **c** but applied to delay 2 of the prospective task. **g** The same as **d** but applied to delay 2 of the prospective task.

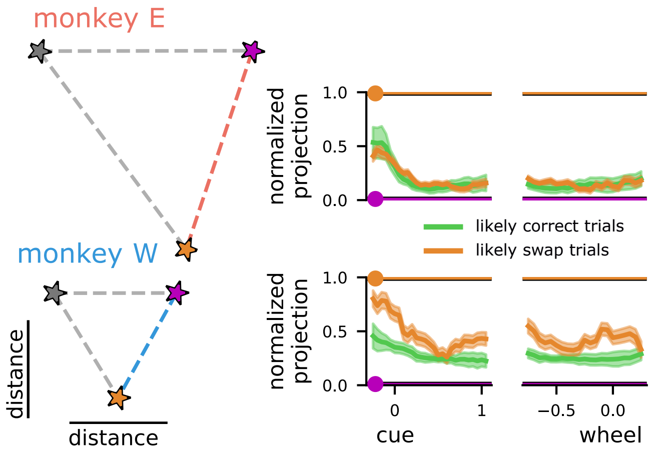

Figure S3: The neural activity projected along the third axis of the triangle in fig. 2 during the second delay period. (left) The geometry of the three hypothesized representations. (right) The projection of neural activity along the dimension connecting the mis-selected colors and mis-interpreted cue representations (purple and orange). (top) Monkey E shows no evidence for cue interpretation errors relative to color selection errors. (bottom) Monkey W shows significant evidence for cue interpretation errors.

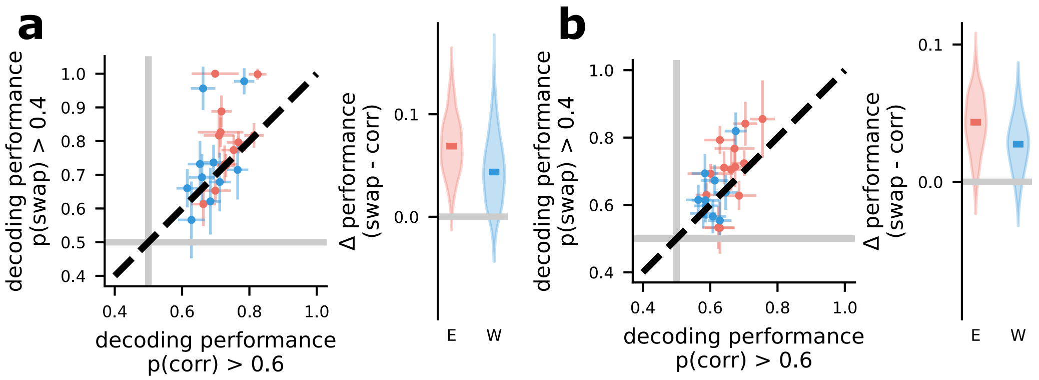

Figure S4: Swap errors are not associated with the encoding of a different number of stimuli in working memory. **a** The performance of a decoder trained to decode the number of stimuli shown during the pre-response wheel period (delay 2) on the retrospective task. (left) The x-axis shows decoding performance on the likely correct trials, while the y-axis shows the same decoder generalized to likely swap trials. (right) Average difference for both monkeys. **b** The same analysis as **a** but for the prospective task.

thus the sources of swap errors in each mode may differ. Since the bimodality and a significant (though small) difference in response time between swap and correct trials exists only in one monkey, we wanted to verify that this does not explain the difference in results between the two monkeys, particularly in the retrospective task.

To address this, we focus on the faster mode by excluding all trials with reaction times longer than 400 ms. Then, we repeat the cross-validated analysis described in the text on both the retrospective (fig. S5) and prospective tasks (fig. S6). This analysis yields results that are qualitatively similar to when all trials are included. While the long response time trials could still be governed by a different process, they are too few to analyze directly here and do not to substantively alter our results when analysing the full set of trials.

References

1. Pratte, M. S. Swap errors in spatial working memory are guesses. *Psychonomic bulletin & review* **26,** 958–966 (2019).

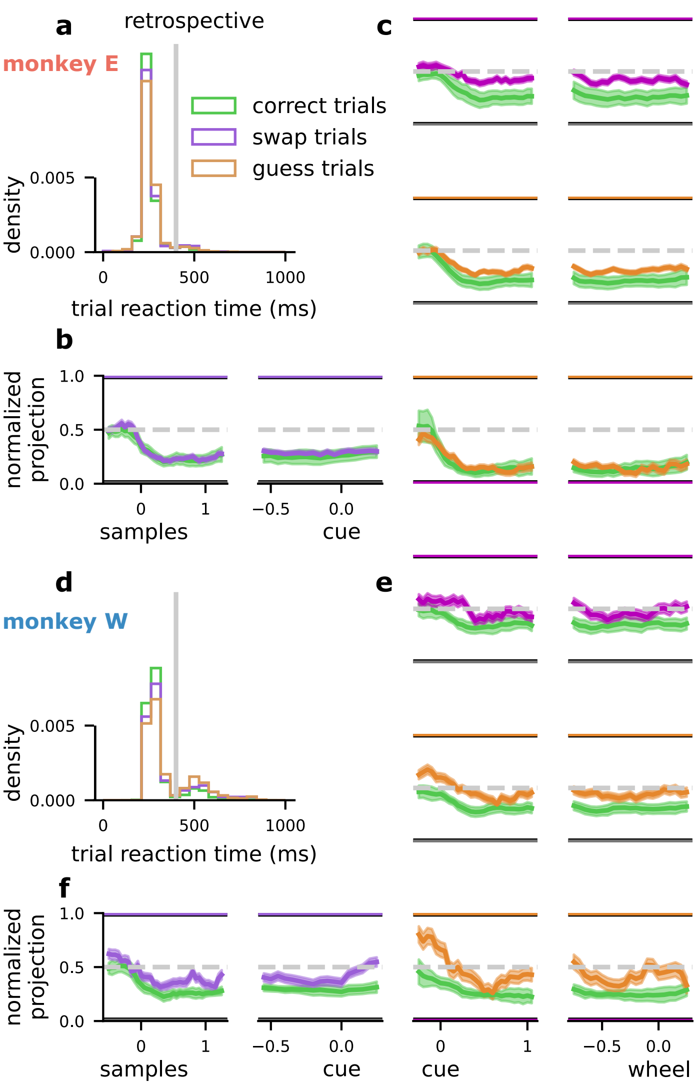

Figure S5: Long reaction time trials do not explain our results on the retrospective task. **a** The distribution of reaction times for monkey E in the retrospective task. All trials with reaction times longer than 400 ms (grey bar) are excluded from the analyses in this figure. **b** The results of the cross-validated analysis for the representation of the colors during the first delay period on likely swap (purple) and likely correct (green) trials. **c** The same as **b** but for the joint representation of the cue and colors during the second delay period. **d, e, f** The same as **a, b, c** but for monkey W.

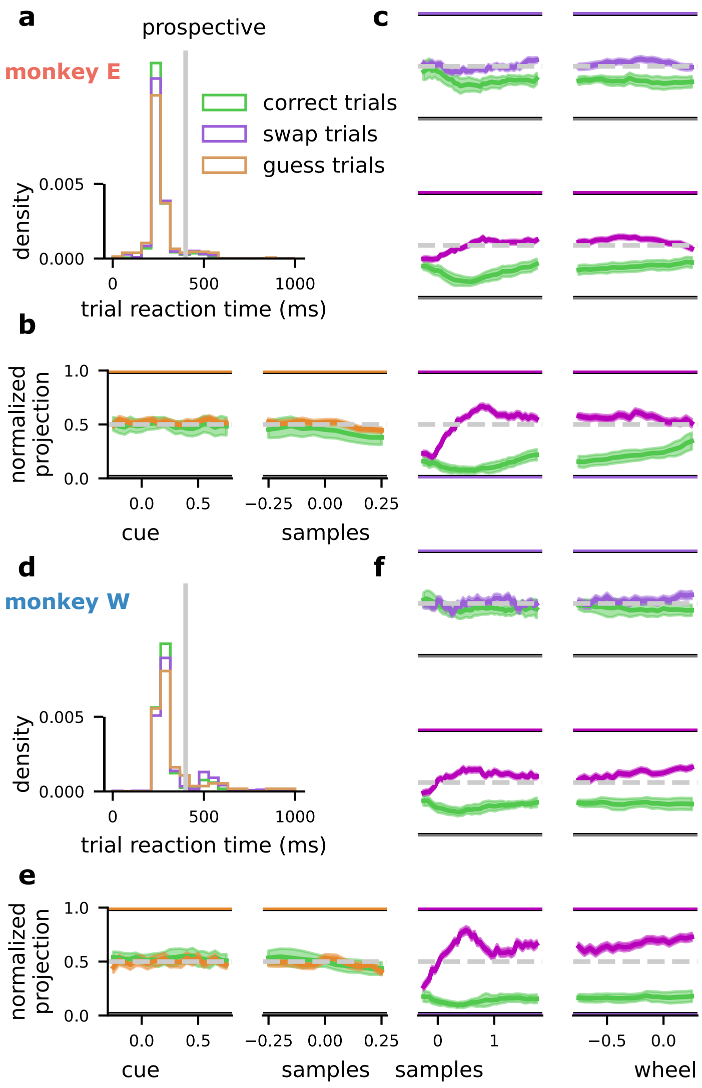

Figure S6: Long reaction time trials do not explain our results on the prospective task. **a** The distribution of reaction times for monkey E in the prospective task. All trials with reaction times longer than 400 ms (grey bar) are excluded from the analyses in this figure. **b** The results of the cross-validated analysis for the representation of the cue during the first delay period on likely swap (yellow) and likely correct (green) trials. **c** The same as **b** but for the joint representation of the cue and colors during the second delay period. **d, e, f** The same as **a, b, c** but for monkey W.

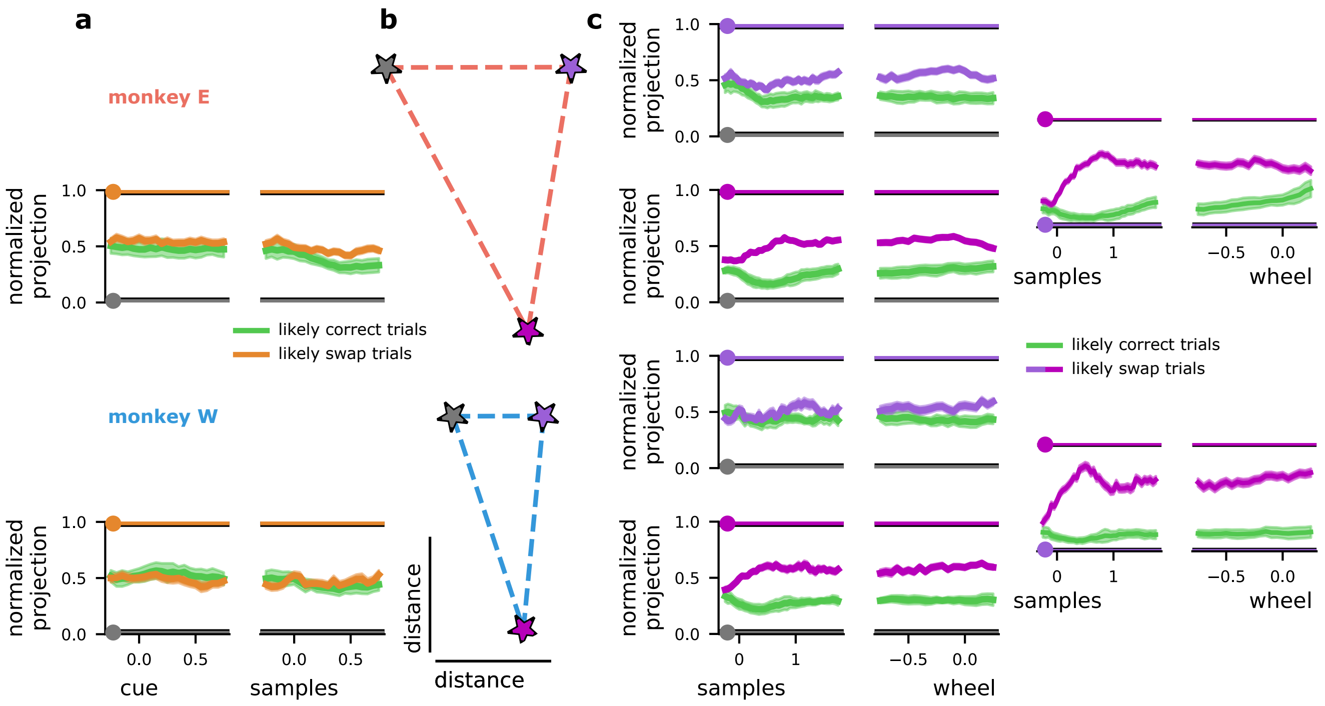

Figure S7: The timecourse of evidence for neural correlates of swap errors in the prospective task. **a** Evidence for cue interpretation errors in monkey E (top) and monkey W (top). **b** The population geometry of hypothesized representations in the second delay period for both monkeys (top and bottom). All distances are significantly greater than zero. **c** (left) Evidence for misbinding (first and third rows) and cue selection (second and fourth rows) in both monkeys (top and bottom blocks). (right) Evidence for cue selection errors in both monkeys.
